# Low frequency of the *TIRAP *S180L polymorphism in Africa, and its potential role in malaria, sepsis, and leprosy

**DOI:** 10.1186/1471-2350-10-65

**Published:** 2009-07-14

**Authors:** Lutz Hamann, Oliver Kumpf, Ron P Schuring, Erkan Alpsoy, George Bedu-Addo, Ulrich Bienzle, Linda Oskam, Frank P Mockenhaupt, Ralf R Schumann

**Affiliations:** 1 Institute of Medical Microbiology and Hygiene, Charité – University Medical Center Berlin, Germany; 2 Institute of Tropical Medicine and International Health, Charité – University Medical Center, Berlin, Germany; 3 Department of Surgery and Surgical Oncology, Robert-Rössle-Klinik, Charité – University Medical Center, Berlin, Germany; 4 KIT (Royal Tropical Institute) Biomedical Research, Amsterdam, The Netherlands; 5 Department of Dermatology and Venerology, Akdeniz University School of Medicine, Antalya, Turkey; 6 Komfo Anoyke Teaching Hospital, School of Medical Sciences, University of Science and Technology, Kumasi, Ghana

## Abstract

**Background:**

The Toll-like receptors (TLRs) mediate innate immunity to various pathogens. A mutation (S180L) in the TLR downstream signal transducer *TIRAP *has recently been reported to be common in Europeans and Africans and to roughly half the risks of heterogeneous infectious diseases including malaria, tuberculosis, bacteremia, and invasive pneumococal disease in heterozygous mutation carriers.

**Methods:**

We assessed the *TIRAP *S180L variant by melting curve and RFLP analysis in 1095 delivering women from malaria-endemic Ghana, as well as in a further 1114 individuals participating in case control studies on sepsis and leprosy in Germany, Turkey and Bangladesh.

**Results:**

In Ghana, the *TIRAP *S180L polymorphism was virtually absent. In contrast, the mutation was observed among 26.6%, 32.9% and 12% of German, Bangladesh and Turkish controls, respectively. No significant association of the heterozygous genotype with sepsis or leprosy was observed. Remarkably, homozygous *TIRAP *180L tend to increase the risk of sepsis in the German study (*P *= 0.04).

**Conclusion:**

A broad protective effect of *TIRAP *S180L against infectious diseases *per se *is not discernible.

## Background

Almost sixty years ago, J. Haldane suggested that malaria had selected hemoglobinopathies to high gene frequencies in malaria-endemic areas due to the protection they confer against this life-threatening disease [1]. This "malaria hypothesis" is now the paradigm of evolutionary selection by infectious diseases, and malaria is considered the strongest known force in this regard [2]. Continuing this early finding a major goal of medical genetics during the last two decades has been to unravel the influence of host's genetics on susceptibility or pathogenesis of common diseases and find specific associations of human genetic variations with these diseases [3,4].

While in case of malaria it is obvious that genetic variations of the erythrocyte – target of the malaria parasite *Plasmodium *– can prevent or modulate disease, host proteins involved in pathogen recognition and defense may also be of pathophysiological importance in malaria [5] and other infectious diseases. The discovery of the toll-like receptor (TLR) system and of central signaling molecules has improved our understanding of host-pathogen interaction in many infectious diseases [6]. Genetic variations within this system have been found and some relate to disease susceptibility and manifestation [7]. *In vitro *and *in vivo *data point to TLRs -2, -4 and -9 to be central for *Plasmodium *recognition and subsequent inflammatory host responses [8-14]. The Toll-interleukin 1 receptor (TIR) domain containing adaptor protein (*TIRAP*, also known as Mal) mediates downstream signaling of TLR-2 and TLR-4, eventually inducing pro-inflammatory responses [15]. Recently, an S180L single nucleotide polymorphism (SNP) of *TIRAP *has been reported to diminish TLR-2 signaling and to occur at a prevalence of some 30% among healthy subjects in the UK and in 2–6% among individuals from West and East Africa. Heterozygosity for this SNP has been claimed to confer protection against malaria and severe malaria to an extent comparable to HbAS, and in addition, to more than halve the risks of bacteremia and tuberculosis in African populations [16]. In an attempt to verify these broad and evolutionary important effects, we screened for *TIRAP *S180L in a Ghanaian population exposed to holoendemic malaria transmission, and examined the role of the SNP in Caucasian and Asian patients affected by sepsis or leprosy.

## Methods

### Study groups

We examined *TIRAP *S180L (rs8177374) in four groups of individuals: i) 1095 delivering women were recruited at the Presbyterian Mission Hospital in hyper- to holoendemic Agogo, Ashanti Region, Ghana in 2000 and in 2006. Diagnosis of placental *Plasmodium falciparum *infection by PCR and malariological indices of the majority of women have been described elsewhere. Briefly, although roughly half of the woman were infected by *Plasmodium falciparum *as evidenced by specific PCR assays, only 4.5% were febrile. Still, anaemia (hemoglobin < 11 g/dL) and low birth weight (< 2500 g) occurred in 33% and 15%, respectively, of woman with live singleton delivery (median age: 25, range 15–47) [17]. ii) 223 patients with sepsis (mean age: 61.4 ± 12.3, male/female: 138/85) and 188 controls without infection (mean age: 62.0 ± 12.5, male/female: 122/66) were recruited at the intensive care unit, Department of Surgery and Surgical Oncology, Robert-Rössle-Klinik, Charité – University Medicine Berlin, Germany, as part of a retrospective case-control study on the influence of different SNPs on sepsis following surgery. Site of infection, relevant microorganisms detected, type of surgery, and infectious complications were recorded (Table 1). Sepsis was defined according to standard criteria [18]. iii) 263 leprosy patients (mean age, 31.1 ± 15.6; male/female, 160/103), from north-west Bangladesh were randomly chosen from the COLEP study [19]. 280 healthy controls were taken from the same study (mean age: 25.2 ± 14.8, male/female: 140/131, data from 9 samples are not available). Patients were classified as paucibacillary (n = 245) or multibacillary (n = 18) according to the 1998 World Health Organization classification for treatment purposes at the Rural Health Program Bangladesh in 2002 and 2003 [20]. iiii) 60 leprosy patients (mean age: 64.0 ± 12.6, male/female: 45/15, lepromatous: n = 50 and tuberculoid leprosy: n = 10) from Turkey, all of Turkish descent, were recruited at the Leprosy Hospital, Elazig, and Akdeniz University, Antalya, Turkey. Diagnosis was based on clinical findings and laboratory tests. Blood smears were analyzed from all patients, skin biopsies were taken from all tuberculoid leprosy patients and most of the lepromatous leprosy patients. *M. leprae *was identified by the detection of acid-fast bacilli and specific histopathological changes. Differential diagnoses, including granulomatous disease, syphilis, tuberculosis, sarcoidosis, and deep fungal infection, were excluded by clinical and laboratory criteria. One hundred asymptomatic control subjects were recruited from the same centers and had neither a family history nor symptoms related to leprosy (mean age: 43.7 ± 10.5, male/female: 45/55).

**Table 1:** Detailed description of sepsis patients

**Cohort (n = 223)**	**Characteristic**	^a^**Ser/Ser** **(n = 170)**	^a^**Ser/Leu** **(n = 43)**	^a^**Leu/Leu** **(n = 10)**
**No (%) of patients**	**Co-existing diseases**			
103 (46.2)	Arterial hypertension	77 (74.8)	22 (21.4)	4 (3.9)
82 (36.8)	Myocardial disease	67 (81.7)	14 (17.0)	1 (1.2)
53 (23.8)	Diabetes	35 (66.0)	14 (26.0)	4 (7.5)
52 (23.3)	Lung pathology	44 (84.6)	6 (11.5)	2 (3.8)
16 (7.2)	Renal pathology	11 (68.8)	4 (25.0)	1 (6.3)
				
**No (%) of patients**	**Type of surgery**			
76 (34.1)	Upper-GI resection	58 (76.3)	16 (21.1)	2 (2.6)
62 (27.8)	Colorectal resection	46 (74.2)	12 (19.4)	4 (6.5)
50 (22.4)	^b^Other abdominal	37 (74.0)	10 (20.0)	3 (6.0)
15 (6.7)	Thoracic	11 (73.4)	3 (20.0)	1 (6.7)
20 (9.0)	Limb and others	18 (90.0)	2 (10.0)	- -
				
**No (%) of infections**	**Type of Infection**			
92 (41.3)	Pneumonia	68 (73.9)	20 (21.7)	4 (4.3)
38 (17.0)	Peritonitis	27 (71.1)	9 (23.7)	2 (5.3)
65 (29.2)	Abscess	52 (80.0)	10 (15.4)	3 (4.6)
4 (1.8)	Urinary tract infections	3 (75.0)	1 (25.0)	- -
24 (10.8)	Other	20 (83.3)	3 (12.5)	1 (4.2)
				
**No (%) of infections**^c^	**Microorganisms**			
129 (57.9)	Gram-negative	97 (75.2)	25 (19.4)	7 (5.4)
83 (37.2)	Gram-positive	60 (72.3)	18 (21.7)	5 (6.0)
15 (6.7)	Fungi	13 (86.7)	2 (13.3)	- -
31 (13.9)	Polymicrobial	19 (61.3)	9 (29.0)	3 (9.8)
24 (10.8)	Clinically diagnosed	15 (62.5)	8 (33.3)	1 (4.2)
				
**No (%) of patients**	**Morbidity and Mortality**			
114 (51.1)	Sepsis without organ dysfunction	90 (78.9)	22 (19.3)	2 (1.8)
109 (48.9)	Severe sepsis and septic shock	80 (73.4)	21 (19.3)	8 (7.3)^d^
28 (12.6)	Mortality ICU	21 (75.0)	6 (21.4)	1 (3.6)
34 (15.3)	Mortality hospital	26 (76.5)	7 (20.6)	1 (2.9)

^a^Ser/Ser indicates wildtype, Ser/Leu indicates heterozygous and Leu/Leu indicates homozygous individuals. ^b^Other abdominal operations: pancreas-, liver- and multiple visceral organ resection. ^c^Numbers might exceed total number of infections due to multiple or no detections. ^d^Increased frequency of Leu/Leu in patients with severe sepsis and septic shock compared to patients without organ dysfunction, 7.3 versus 1.8%, *P *= 0.1. No significant differences were observed by comparing patient characteristics. Abbreviation: ICU: Intensive Care Unit.

### Ethical approval

The study protocols were reviewed and approved by the Committee on Human Research Publication and Ethics, School of Medical Sciences, University for Science and Technology, Kumasi, Ghana, and by the human subject review committees at Humboldt-University Berlin, Germany, and Akdeniz University, Antalya, Turkey, and the institutional guidelines were followed in conducting this study. For the Bangladesh study, written approval was granted by the Ethical Review Committee of Bangladesh Medical Research Council (BMRC/ERC/2001–2004/799 and BMRC/ERC/2004–2007/120.

### Sample size calculations

For this exploratory survey, no definite sample size calculation was performed. However, considering published average prevalences of *TIRAP *S180L of 30% in Caucasians, Asians and 4% in Africans [16], and 33% in Bangladesh, this study had a power of 80% at a 95% confidence level to display risk reductions (odds ratios) of 0.27, 0.50, 0.22, and 0.56 for malaria, sepsis, leprosy (Turkey) and leprosy (Bangladesh), respectively.

### Genotyping

DNA was extracted from blood by standard techniques. Genotyping for *TIRAP *S180L was carried out by melting curve analysis employing the Lightcycler 480 device (Roche Diagnostics, Mannheim, Germany) using the following primers and probes: sense primer: GCCAGGCACTGAGCAGTAGT, antisense primer: GTGGGTAGGCAGC-TCTTCTG, anchor probe: Red640-GATGGTGCAGCCCTCGGCCCC, and sensor probe: AGGCCCAACAGCAGGG-FL. The melting peaks are at 53°C and 62°C for the wildtype and the mutated sequences, respectively. Due to secondary structures and allele biased amplification within the region of this SNP, analysis of heterozygous genotypes may sometimes result in false homozygous results. Therefore, all mutated samples were reanalysed by conventional RFLP method described by Khor et al. [16]. Genomic DNA was amplified with following primers: CTCCAGGGGCCGAGGGCTGCACCATCCCCATGCTG and TACT-GTAGCTGAATCCCGTTCC. The resulting PCR product was digested with BstXI and analysed by gel electrophoresis.

### Statistical analysis

Comparison of proportions of heterozygous and wild type individuals was done by χ^2 ^or Fisher's exact test, as applicable.

## Results

The prevalence of *TIRAP *S180L among German controls of 26.6% (allele frequency, 19.2%) closely matched the reported figure from the United Kingdom [16]. Surprisingly, however, we detected the heterozygous *TIRAP *S180L variant in only one (0.1%) out of 1095 individuals from malaria holoendemic Ghana. This asymptomatic woman had a placental *P. falciparum *infection with a mean of 28 parasites/microscopic high power field, and prematurely delivered a child of low birth weight. The mere absence of the SNP in more than 500 *P. falciparum *non-infected women rendered statistical analysis meaningless (Table 2).

**Table 2:** Frequency of the *TIRAP-*S180L variant in different populations

			No. of individuals with genotype (%)			
				
Population and disease	No.	Variant Allele Frequency (%)	Ser/Ser	Ser/Leu	Leu/Leu	*P *value^a^
Ghana, delivering women						
*P. falciparum *positive	541	0.1	540 (99.8)	1 (0.2)	0 (0)	0.49
*P. falciparum *negative	554	0	554 (100)	0 (0)	0 (0)	
Germany						
Sepsis patients	223	18.1	170 (76.2)	43 (19.3)	10 (4.5)	0.19
Controls	188	19.2	138 (74.4)	48 (25.5)	2 (1.1)^b^	
Bangladesh						
Leprosy patients	263	16.3	186 (70.7)	68 (25.9)	9 (3.4)	0.22
Controls	280	17.2	188 (67.1)	88 (31.5)	4 (1.4)	
Turkey						0.11
Leprosy patients	60	10.8	47 (78)	13 (21)	0 (0)	
Controls	100	6.5	88 (88)	11 (11)	1 (1)	

^a ^Comparison of proportions of heterozygotes and wildtype individuals by χ^2 ^or Fisher's exact test, as applicable. ^b^*P *value = 0.04, for comparison of homozygous 180L individuals and other individuals. Differences of allele frequencies are not significant.

In the German sepsis study, the heterozygous *TIRAP *S180L genotype was frequent but there was no significant association with the risk of developing sepsis. Nevertheless, heterozygosity was slightly more frequent in controls than in patients, 25.5% versus 19.3%, respectively (*P *= 0.19, Table 2), potentially indicating some advantage when the heterozygous genotype was present. In contrast, the homozygous *TIRAP *180L genotype was significantly more frequent in patients, (4.5 versus 1.1%, *P *= 0.04, Table 2), indicating this genotype to potentially be a risk factor for developing sepsis. This is also being reflected by a trend showing a slightly increased frequency of the homozygous *TIRA*P 180L within the patient group of severe sepsis and septic shock compared to septic patients without organ dysfunction (7.4 versus 1.7%, *P *= 0.1, Table 1).

Both leprosy studies showed no significant association either of heterozygosity or homozygosity with leprosy (Table 2). Again, we found a slightly enhanced frequency of heterozygosity in controls from Bangladesh compared to leprosy patients, 31.4% versus 25.9% (*P *= 0.22), also potentially indicating some degree of advantage of the heterozygous mutation for leprosy. The small study from Turkey showed controversial results. We observed heterozygous *TIRAP *S180L in 11% of control individuals, but more frequently, 22%, in leprosy patients (Table 2). However, the sample number of this study is too low for significant conclusions.

## Discussion

We here report the virtual absence of a putatively malaria-protective SNP in an area of holoendemic malaria transmission in West Africa. This not only contrasts the published prevalence among African populations of 2–6% [16], also, and more importantly, it argues against clinical-epidemiological relevance in malaria and against the hypothesis that malaria has exerted significant positive selective pressure on the presence of this SNP. According to Haldane's malaria hypothesis [1,2], higher frequencies of this SNP than reported and observed here should be expected in highly malaria-endemic areas considering the postulated protective effect against this disease of 42–96% [16]. We are at loss to explain the discrepancy between the reported prevalences of 5.9 and 3.4% among control populations in such distantly located African countries as The Gambia and Kenya and the 0.1% we found in Ghana. Nevertheless, the latter figure – particularly when facing the 15–30% in Caucasians – strongly argues against an expected positive selection of a malaria-protective trait in Africa. The absence of homozygosity for *TIRAP *180L in the African studies could suggest a state of balanced polymorphism due to a yet unknown disadvantage in homozygotes and acting against relevant allele frequencies. A homozygous disadvantage has been claimed for Caucasians with respect to a slightly increased risk for invasive pneumococcal disease and pneumococcal empyema [16]. One theoretical limitation of the present study is that we examined predominantly asymptomatic women with or without placental *P. falciparum *infection. Thus, potential protective features of the SNP could be missed in individuals with non-severe malaria. However, in The Gambia the risk reductions in terms of severe malaria and "general malaria" were very similar [16]. Ultimately, the extremely low prevalence of heterozygous *TIRAP *S180L in Ghana, where malaria endemicity is among the highest in the world argues against attempts to replicate its putatively protective effect: Given the observed prevalence of heterozygosity of 0.1%, a study able to confirm the reported risk reduction would need to comprise several tens of thousands of individuals to have sufficient statistical power. This, in turn, is even another argument against the presumed clinical relevance of this mutation in Africa.

Heterozygosity for *TIRAP *S180L has been suggested to be protective not only against malaria but also against bacterial infections including invasive pneumococcal disease, pneumococcal empyema, overall and pneumococcal bacteremia, and tuberculosis [16]. In our study populations from Germany, Bangladesh, and Turkey we found allele frequencies of 19, 17, and 6.5%, respectively. However, we only found very limited evidence for heterozygosity being protective in both, sepsis and leprosy patients. Likewise, within the subgroup of sepsis patients suffering from pneumonia no significant protection of heterozygosity could be observed (Table 1). In the sepsis study, the proportion of heterozygous *TIRAP *S180L genotype seem to be influenced by co-existing diseases such as Diabetes and renal pathology, however, there is no significant difference (Table 1). In contrast, we found at borderline significance an increased frequency of homozygosity in sepsis patients (p = 0.04, Table 2). This is in line with the data reported by Khor et al. [16], who also showed an increased, but not significant, frequency of homozygosity in invasive pneumococcal disease and pneumococcal empyema. However, larger studies are needed to verify whether heterozygosity is protective or homozygosity is a risk factor. Remarkably also, in a recent study from Ghana, Russia and Indonesia, *TIRAP *S180L also failed to show any association of heterozygosity with tuberculosis in any population. Of note, this study also found a very low allele frequency for the *TIRAP *S180L of only 0.08% in Ghana [21].

## Conclusion

While the TLR system has been shown to be critically involved in susceptibility to and manifestation of malaria, we found a very low frequency of the S180L mutation in the TLR downstream mediator *TIRAP *in a highly endemic region arguing against a major role of this SNP in malaria. In our study populations with a relative high prevalence of the *TIRAP *S180L, we found a trend but no significant protection of heterozygosity in sepsis and leprosy. In contrast, in sepsis *TIRAP *180L homozygosity appears to be a risk factor for disease. Taking together our data and those of Khor et al. [16], we suggest that there might be a slight protective effect of heterozygous *TIRAP *S180L in several diseases. At the same time homozygous *TIRAP *180L appears to be a risk factor for the same diseases. The specific role of *TIRAP *S180L needs further investigation.

## Competing interests

The authors declare that they have no competing interests.

## Authors' contributions

Samples were collected by EA, OK, RPS, LO, GB-A, and FPM. Genotyping was performed by LH. Statistics were done by FPM and RPS. LH, UB, FPM, and RRS conceptualized and designed the study. LH, FPM, and RRS wrote the manuscript. All authors read and approved the final manuscript.

## Pre-publication history

The pre-publication history for this paper can be accessed here:


